# Smoking at time of diagnosis and breast cancer-specific survival: new findings and systematic review with meta-analysis

**DOI:** 10.1186/bcr3646

**Published:** 2014-04-19

**Authors:** Sylvie Bérubé, Julie Lemieux, Lynne Moore, Elizabeth Maunsell, Jacques Brisson

**Affiliations:** 1 Centre des maladies du sein Deschênes-Fabia, CHU de Québec, Hôpital du Saint-Sacrement, 1050 Chemin Sainte-Foy, Québec, Qc G1S 4 L8, Canada; 2 Centre de recherche du CHU de Québec, Hôpital du Saint-Sacrement, Québec, Canada; 3 Département de médecine, Faculté de médecine, Université Laval, Québec, Canada; 4 Département de médecine et Service d’hémato-oncologie, CHU de Québec, Québec, Canada; 5 Département de médecine sociale et préventive, Faculté de médecine, Université Laval, Québec, Canada; 6 Unité de traumatologie-urgence-soins intensifs, CHU de Québec, Québec, Canada

## Abstract

**Introduction:**

In women with breast cancer who smoke, it is unclear whether smoking could impair their survival from the disease.

**Methods:**

We examined the relation of smoking at diagnosis to breast cancer-specific and overall survival among 5,892 women with invasive breast cancer treated in one Canadian center (1987 to 2008). Women were classified as never, former or current smokers. Current smokers were further classified according to total, intensity and duration of smoking. Deaths were identified through linkage to population mortality data. Cox proportional-hazards multivariate models were used. A systematic review with meta-analysis combines new findings with published results.

**Results:**

Compared with never smokers, current smokers at diagnosis had a slightly, but not statistically significant, higher breast cancer-specific mortality (hazard ratio = 1.15, 95% confidence interval (CI): 0.97 to 1.37). Among current smokers, breast cancer-specific mortality increased with total exposure to, intensity and duration of smoking (all *P*_trend_ <0.05). Compared to never smokers, breast cancer-specific mortality was 32 to 56% higher among heavy smokers (more than 30 pack years of smoking, more than 20 cigarettes per day or more than 30 years of smoking). Smoking at diagnosis was associated with an increased all-cause mortality rate. A meta-analysis of all studies showed a statistically significant, 33% increased mortality from breast cancer in women with breast cancer who are smokers at diagnosis compared to never smokers (hazard ratio = 1.33, 95% CI: 1.12 to 1.58).

**Conclusions:**

Available evidence to date indicates that smoking at diagnosis is associated with a reduction of both overall and breast cancer-specific survival. Studies of the effect of smoking cessation after diagnosis on breast cancer-specific outcomes are needed.

## Introduction

Women with breast cancer are eager to do what they can to improve their prognosis [1]. However, apart from weight control and regular physical activity [2,3], there are few lifestyle changes that are known to improve the prognosis of the disease.

Smoking cessation is perhaps a lifestyle choice that women with breast cancer could make to improve their prognosis. Numerous studies show that smoking is associated with a reduction of overall survival among smokers who are diagnosed with breast cancer [4-14]. This decreased survival is attributable, at least in part, to increased mortality from causes other than breast cancer that are associated with smoking.

Some studies have suggested that smoking at diagnosis may also be associated with increased mortality from breast cancer itself. However, while some studies observed such an increase in post-diagnosis breast cancer-specific mortality in smokers [4,5,8,11,12,15], this increase was statistically significant in five studies [4,5,8,12,15] but some studies showed little or no association between smoking and breast cancer survival [6,9,16].

Clarification of whether smoking could affect breast cancer-specific mortality is important as it would shape survivorship health messages to the large population of smokers who are diagnosed with the disease. This study examines the relation of smoking at time of breast cancer diagnosis to long-term breast cancer-specific survival and overall survival, in a large cohort of women diagnosed with and treated for invasive breast cancer between 1987 and 2008 at one Breast Center in Quebec City, Canada. In addition, a systematic review with meta-analysis is also reported combining the present findings with all available published results on smoking and breast cancer-specific survival.

## Methods

### Smoking at diagnosis and survival: new findings

#### Study population

From January 1987 through May 2008, 6,377 women were diagnosed with primary invasive breast carcinoma and received their initial treatments at the ‘Centre des maladies du sein Deschênes-Fabia - CMSDF.’ This analysis is based on the 5,892 (92.4%) cases with known smoking status at diagnosis; most cases are whites of European descent. Since data were collected from patient’s charts without contact with participants, access to charts was authorized by the Director of professional services and the study was approved by the Research Ethics Review Board of our center (Centre hospitalier affilié universitaire de Québec, Quebec, Quebec, Canada) without the need for individual patient’s consent.

#### Data collection

Information on smoking at diagnosis and several characteristics of women was extracted from a registry held by the CMSDF.

At diagnosis, women were asked whether they had ever smoked cigarettes and, if so, the usual number of cigarettes smoked daily, for how many years they had smoked and whether they were still smoking. Other characteristics collected include year of diagnosis, age, height and weight, obstetrical and gynecological history, first degree family history of breast cancer and alcohol consumption. The presence of comorbid conditions [17,18] in the six-month period preceding the diagnosis of breast cancer, including diabetes and its chronic complications, was assessed by record linkage with MED-ECHO files, a comprehensive population-based hospitalization and day-surgery data base. This database includes one principal diagnosis and up to 15 secondary diagnoses at the time of any hospitalization or day-surgery.

Characteristics of the disease including estrogen and progesterone receptor status, histological grade [19], tumor size, and regional or distant involvement were also recorded in the Breast Center registry as well as treatments including initial surgical treatments, neoadjuvant and adjuvant therapy (endocrine therapy, chemotherapy and, more recently, the monoclonal antibody Herceptin®).

Information on women’s vital status was determined up to 31 October 2008 (the termination date for the present analysis), by linking personal identifying information to the database of beneficiaries of the Quebec universal health insurance system, and to the Quebec mortality database held by the ‘Institut de la statistique du Québec, ISQ.’ Causes of death, which were extracted from the ISQ database, were coded based on the International Classification of Diseases; breast cancer deaths were those coded 174.9 (Ninth revision until 1999) and C50.9 (Tenth revision thereafter).

#### Statistical analysis

The date of diagnosis was defined, in order of priority, as the date of: 1) the first histopathological confirmation of the malignancy (available in 98.4% of cases), 2) the first positive cytology, or 3) the first clinical investigation (mainly mammography) showing malignancy. For most women (n = 5,868), person-years of follow-up were calculated from the date of diagnosis of breast cancer until the earliest of date of death or 31 October 2008. Women who did not match with administrative databases (n = 13) and those identified as being no longer covered by the Quebec health insurance (n = 11) were censored at the date of their last visit at the Breast Center or at the date of interruption of the insurance coverage, respectively.

For descriptive purposes, breast cancer-specific and overall survival rates following the diagnosis were calculated by the actuarial method with one-year time intervals using death from breast cancer or of any cause, respectively, as endpoint. Deaths of unknown cause (14 cases, representing 1% of all deaths) were considered to be due to causes other than breast cancer. Baseline characteristics were presented according to smoking status.

Cox proportional hazards models were used to calculate hazard ratios (HR) and 95% confidence intervals (CI) of death from breast cancer (breast cancer-specific mortality HRs) and death from any cause (overall mortality HRs). HRs (and 95% CIs) were calculated for smoking categories (never, former, current) with never smokers as the reference group. Current smokers were further classified according to categories of total exposure to smoking, computed as the product of duration of smoking and mean number of packs (20 cigarettes) smoked per day (≤15, >15 to 30, >30 pack years), categories of intensity of smoking (≤10, 10 to 20, >20 cigarettes/day) and categories of duration of smoking (≤20, 21 to 30, >30 years). In these same Cox models, *P* values for trend in HRs according to total, intensity and duration of smoking are based on the Wald test of the linear contrast between the four categories compared (never smokers and the three categories of current smokers) [20]. Former smokers were excluded from this contrast. Finally, there was no indication that the proportional hazards assumption was violated whether based on the inspection of plots of log(−log(survival)) versus log of survival time curves or on statistical tests [21].

In all multivariate Cox models, adjustments were made for a large set of factors known or suspected to confound the relation of smoking to breast cancer risk [22-25] or to prognosis of breast cancer [2,25-28]: year of diagnosis, age at diagnosis, age at menarche, parity, menopausal status, current hormone replacement therapy use, first degree family history of breast cancer, estrogen and progesterone receptor positivity, histological grade, size of the tumor, regional or distant involvement, locoregional treatment, neoadjuvant therapy, adjuvant endocrine therapy and adjuvant chemotherapy. Adjuvant treatment with Herceptin (trastuzumab) was not considered in the present analysis: it has been available only since 2005 in our center, and was used by less than 150 cases participating in the present analysis. However, trastuzumab has been found to offer similar benefits to smokers and non-smokers [29]. Multiple imputation techniques [30,31] were used to handle missing data on potential confounders. For each of the two outcomes (breast cancer-specific mortality and overall mortality), 50 imputed datasets were generated and results were combined using the PROC MI and PROC MIANALYZE commands in SAS (version 9.3) with appropriate correction for variance. The data imputation models included the outcome variables (survival time, vital status and cause of death, and an interaction term between survival time and outcome), smoking status and smoking exposure variables among smokers (total, intensity and duration), all potential confounders above, alcohol use, and a few additional variables (weight and height, clinical tumor size). Sensitivity analyses were performed. First, results obtained in the entire cohort of 5,892 women with multiple imputation were compared to those obtained with the subset of 4,334 subjects for whom all data (except alcohol use) were available (complete case analysis [32]). Second, analyses were performed on the entire cohort of 5,892 women using indicator variables for missing data on confounders. Third, since alcohol use was not collected among women diagnosed in 1999 to 2003, and was thus missing on nearly half the cases, confounding by alcohol use (yes, no) was assessed in the entire cohort (n = 5,892) with multiple imputation, and also in the subset of cases (n = 2,315) with complete data including alcohol use and in the analysis using missing indicator variables to handle missing data on the other confounders (n = 3099). Fourth, an analysis was done to assess the effect of adjustment for different covariates. Models additionally adjusted for body mass index and diabetes were computed. Also, smoking has been hypothesized to increase breast cancer-specific mortality by promoting more aggressive tumors [8,9,11,16] or because smokers experience delay in diagnosis and treatment of their disease [9,11]. Thus, such characteristics could be viewed as intermediate factors in the pathway relating smoking to breast cancer-specific mortality. The effect of adjustment for individual co-variable or groups of co-variables was investigated by comparing the smoking HR obtained from a fully-adjusted model to the smoking HR obtained from a model that excluded one co-variable or a group of co-variables (for instance exclusion from a model of grade, hormonal receptor status, tumor size or stage at diagnosis individually or exclusion of grade and hormonal receptor status (representing the biology of the tumor), or exclusion of tumor size and stage at diagnosis (representing the extent of disease at diagnosis) or exclusion of all these tumor related co-variables (representing tumor characteristics). Finally, interactions of smoking status with age at diagnosis, menopausal status, body mass index, ER/PR status, regional or distal involvement and treatments were assessed.

Statistical significance was based on two-sided *P* values. All statistical analyses were carried out by using the Statistical Analysis System (SAS Institute, Cary, NC, USA version 9.3).

### Systematic review and meta-analysis of published findings

Published cohort studies on the relation of smoking to breast cancer-specific survival were identified from PubMed (up to 4 July 2013) using the medical subject headings (MeSH) terms ("Breast Neoplasms"[Majr:NoExp]) AND ((((("Survival"[Mesh]) OR "Mortality"[Mesh]) OR "Survival Rate"[Mesh]) OR "Disease-Free Survival"[Mesh]) OR "Survival Analysis"[Mesh] OR Mortality OR Survival) AND ("Habits"[Mesh]). Only English and French language papers were eligible for inclusion. Bibliographies of retrieved papers were also searched. The relevance of each of the papers identified was judged independently by two of us (SB and JB) based on the title or abstract and then the retrieved paper. For all retained papers, detailed data were extracted including HR (95% CI) for current smoking as compared to never smokers, adjustment variables and variables used for stratified analyses. The quality of studies was assessed using the Newcastle-Ottawa Scale, a standardized tool [33].

The meta-analysis was performed using REVMAN statistical software (version 5.1) [34]. Summary HRs and 95% CIs were estimated using the random-effects model. Heterogeneity between studies was assessed using the I^2^ statistic and the Cochran Q test.

Sensitivity analysis was performed to examine the effect on both the summary HRs and the heterogeneity assessment of excluding studies most likely responsible for heterogeneity between studies.

## Results

### Smoking and survival in the cohort

The 5,892 women with invasive breast cancer included in the analyses accumulated 41,255 person-years of follow-up; 53.9% of breast cancer cases were followed for at least 5 years, 25.7% at least 10 years, and 11.4% at least 15 years (maximum: 22 years). During the follow-up period, 1,408 deaths were documented, of which 953 (67.7%) were from breast cancer, 441 (31.3%) from other causes and 14 (1.0%) from unknown causes. The five-year and ten-year breast cancer-specific survival estimates were 87% and 79%, respectively, and five-year and ten-year overall survival estimates were 83% and 71%, respectively.

At diagnosis, 60% of women reported having never smoked, 22% were former smokers and 18% were current smokers (Table 1). Current smokers appeared to be younger, leaner and more likely to be alcohol drinkers compared with never smokers. Characteristics of the disease and treatment differed little according to smoking status.

**Table 1:** Characteristics of women with invasive breast cancer according to smoking status at diagnosis (1987 to 2008)

	**Smoking status at time of diagnosis**			
	**Current (n = 1,079)**	**Former (n = 1,303)**	**Never (n = 3,510)**	**Total (n = 5,892)**
Characteristics of women				
Age (years), mean (SD)	52.8 (11.5)	56.1 (11.3)	59.3 (13.1)	57.4 (12.7)
Body mass index^b^ (kg/m^2^), mean (SD)	24.0 (4.6)	25.5 (5.0)	25.1 (4.7)	25.0 (4.8)
Age (years) at menarche^b^, mean (SD)	12.8 (1.8)	12.8 (1.7)	12.9 (1.6)	12.9 (1.7)
Parity^b^, mean (SD)	1.9 (1.8)	1.9 (1.8)	2.3 (2.2)	2.1 (2.1)
Postmenopausal women^b^ (%)	60.5	67.6	73.6	69.8
Age (years) at menopause^b^, mean (SD)	45.4 (6.9)	46.3 (6.8)	47.1 (6.4)	46.7 (6.6)
Hormone replacement therapy current use^b^ (%)	17.1	17.2	17.5	17.3
Family history of breast cancer^a, b^ (%)	23.2	24.5	25.6	24.9
Diabetes^b^ (%)	1.9	1.9	2.6	2.3
Current alcohol intake^b^ (%)	39.5	47.3	27.3	34.2
Characteristics of the disease and treatment				
Tumor size (mm)^b^, mean (SD)	22.9 (17.7)	22.7 (17.5)	23.3 (18.3)	23.1 (18.0)
Number of axillary nodes examined^b, c^, mean (SD)	12.4 (7.8)	11.1 (8.2)	11.2 (7.7)	11.4 (7.9)
Positive estrogen receptors (%)	73.3	77.7	73.4	74.1
Positive progesterone receptors (%)	56.1	56.9	52.2	53.6
Well differentiated histological grade^b^ (%)	24.5	27.8	26.0	26.1
Regional involvement (%)	39.4	36.4	36.1	36.8
Distant metastasis at diagnosis (%)	3.2	2.5	3.2	3.0
Breast-conserving surgery^d^ (%)	68.3	72.1	67.4	68.6
Axillary surgery^d^ (%)	89.4	90.9	85.0	87.1
Neoadjuvant therapy^d^ (%)	5.4	6.7	6.4	6.3
Adjuvant radiotherapy^b, d^ (%)	75.9	79.8	71.4	74.1
Adjuvant chemotherapy^b, d^ (%)	49.0	48.6	41.4	44.4
Adjuvant endocrine therapy^b, d^ (%)	60.8	65.2	64.7	64.1

^a^Mother, sister and daughter. ^b^Missing information in less than 5% of women, except for histological grade (14%), pathological tumor size (6.1%), and alcohol use (47.4%). ^c^In 5,132 women who had an axillary surgery or a sentinel node biopsy. ^d^In 5,713 women with nonmetastatic breast cancer.

Based on unadjusted models, there was no statistically significant association between smoking at diagnosis (status, total exposure, intensity or duration) and breast cancer-specific mortality (Table 2). The effects of total smoking exposure, intensity and duration of smoking became more apparent after taking age at diagnosis into account. Based on multivariate adjusted models, breast cancer-specific mortality was 4% (HR = 1.04, 95% CI: 0.88 to 1.24) and 15% (HR = 1.15, 95% CI: 0.97 to 1.37) higher in former and current smokers at diagnosis, respectively, compared to never smokers at diagnosis, but this association was not statistically significant (*P*_association_ = 0.30). However, there was a trend for increased HRs with increasing pack years of smoking at diagnosis (*P*_trend_ = 0.002), with increasing number of cigarettes smoked daily (*P*_trend_ = 0.03) and with increasing duration of smoking (*P*_trend_ = 0.004) compared to never smokers. Among current smokers at diagnosis, breast cancer-specific mortality was 52% higher (HR = 1.52, 95% CI: 1.15 to 2.00) for women with more than 30 pack years of smoking, 32% higher (HR = 1.32, 95% CI: 1.02 to 1.70) among those smoking more than 20 cigarettes per day and 56% higher (HR = 1.56, 95% CI: 1.19 to 2.05) among those smoking for more than 30 years, compared to never smokers. An increase in the breast cancer-specific mortality rate of 52% among women with more than 30 pack years of smoking as compared with never smokers would be associated with an absolute reduction of 9.1% in the ten-year breast cancer survival in this group.

**Table 2:** Breast cancer-specific mortality according to smoking exposure at time of diagnosis among 5,892 women with invasive breast cancer (1987 to 2008)

**Smoking status**			**Breast cancer-specific mortality**		
	**Number of**		**Crude HR (95% CI)**	**Age**^**a**^**-adjusted HR (95% CI)**	**Adjusted**^**b **^**HR (95% CI)**
	**Women**	**Deaths**			
Never^c^	3,510	583	1.00	1.00	1.00
Former	1,303	185	0.94 (0.79 to 1.11)	0.99 (0.84 to 1.17)	1.04 (0.88 to 1.24)
Current^d^	1,079	185	0.99 (0.84 to 1.16)	0.99 (0.83 to 1.17)	1.15 (0.97 to 1.37)
≤15 pack years	232	40	0.82 (0.60 to 1.13)	0.73 (0.53 to 1.01)	0.92 (0.66 to 1.28)
>15 to ≤30 pack years	278	54	1.06 (0.80 to 1.40)	1.05 (0.79 to 1.39)	1.23 (0.92 to 1.64)
>30 pack years	273	58	1.20 (0.91 to 1.57)	1.35 (1.03 to 1.78)	1.52 (1.15 to 2.00)
*P*-value, test for trend^e^			0.08	0.004	0.002
≤10 cigarettes/day	287	36	0.75 (0.54 to 1.05)	0.73 (0.52 to 1.02)	0.97 (0.69 to 1.37)
>10 to ≤20 cigarettes/day	395	75	1.05 (0.83 to 1.34)	1.06 (0.83 to 1.35)	1.13 (0.88 to 1.45)
>20 cigarettes/day	337	70	1.17 (0.91 to 1.50)	1.19 (0.92 to 1.53)	1.32 (1.02 to 1.70)
*P*-value, test for trend^e^			0.06	0.04	0.03
≤20 years	191	44	1.06 (0.78 to 1.44)	0.87 (0.63 to 1.19)	1.05 (0.76 to 1.45)
21 to 30 years	258	49	0.93 (0.69 to 1.24)	0.92 (0.68 to 1.24)	1.06 (0.78 to 1.44)
>30 years	340	61	1.11 (0.85 to 1.45)	1.30 (1.00 to 1.70)	1.56 (1.19 to 2.05)
*P*-value, test for trend^e^			0.67	0.07	0.004

^a^HRs adjusted for age at diagnosis (≤39, 40 to 49, 50 to 59, 60 to 69, ≥70 years). ^b^HRs adjusted for age at diagnosis (≤39, 40 to 49, 50 to 59, 60 to 69, ≥70 years), year of diagnosis (≤1994, 1995 to 1999, 2000 to 2004, ≥2005), age at menarche (≤11, 12, 13, ≥14 years), parity (0, 1, 2, ≥3), menopausal status (premenopausal, postmenopausal), current hormone replacement therapy use (yes, no), first degree family history of breast cancer (yes, no), estrogen and progesterone receptors positivity (positive, negative, not investigated), histological grade (well, moderately, poorly differentiated), size of the tumor (≤10, 11 to 20, 21 to 30, 31 to 40, >40 mm, no pathological investigation), regional or distant involvement (node-negative, 1 to 3 positive nodes, ≥4 positive nodes, unknown nodal involvement, distant metastases), locoregional treatment (mastectomy, breast-conserving surgery, no surgery), neoadjuvant therapy (yes, no), adjuvant endocrine therapy (yes, no), adjuvant chemotherapy (yes, no). ^c^Reference category. ^d^Among the 1,079 current smokers, number of pack years of smoking, number of cigarettes smoked and duration of smoking were unknown for 296, 60 and 290 women, respectively. ^e^*P* for trend in hazard ratios comparing current smokers further categorized based on number of pack years of smoking, number of cigarettes smoked daily or duration of smoking, to never smokers. HR, hazard ratio; CI, confidence interval*.*

With respect to our sensitivity analyses, results based on complete case analysis or on the analysis using missing indicator variables for confounders were similar or differed only slightly from those obtained with multiple imputation. For instance, when compared to never smokers, the HRs for those who had more than 30 pack years of smoking were 1.52 (1.15 to 2.00) in the main analysis, 1.45 (1.05 to 2.02) in the complete cases analysis (see Additional file 1), and 1.52 (1.15 to 2.01) (see Additional file 2) in the analysis with missing indicator variables. In the main analysis with multiple imputation, in the subset of cases (n = 2,315) with complete data including alcohol use, or in the analysis using indicator variables for missing data on confounders other than alcohol (n = 3,099), further adjustment for alcohol use increased rather than decreased multivariate adjusted HRs relating smoking to breast cancer-specific mortality by 2% or less. Sensitivity analyses of confounding effects suggest that body mass index and diabetes had no confounding effects, while tumor characteristics had notable confounding effects. Multivariate adjusted HRs relating smoking at diagnosis to breast cancer-specific mortality from models that included characteristics of the tumor (receptor status, grade, size of tumor and stage) were generally similar or slightly higher than those that excluded these characteristics individually from the models. However, when all these characteristics were excluded from the model, the strength of the association was reduced. For instance, exclusion of all such tumor characteristics from the full model decreased the HR associated with current smoking from 1.15 to 1.02 (−11.3%).

Interactions of smoking status with age at diagnosis, menopausal status, body mass index, ER/PR status, regional or distal involvement and treatments were assessed, and none of these interaction tests was statistically significant both in complete case analysis and in the analysis with indicator variables for missing data on confounders.

The associations between smoking exposure variables and all-cause mortality were stronger than those with breast cancer-specific mortality and were statistically significant (all *P*_association_ ≤0.0001) (Table 3). For instance, all-cause mortality was 17% and 38% higher in former and current smokers at diagnosis, respectively, as compared with never smokers. All-cause mortality also increased with increasing pack years of smoking (*P*_trend_ <0.0001), increasing intensity (*P*_trend_ = 0.0001) and increasing duration of smoking (*P*_trend_ <0.0001) among current smokers at diagnosis, as compared with never smokers. Compared to the main analysis, HRs were only slightly lower in the complete case analysis (see Additional file 3) and were similar in the analysis with missing indicators (see Additional file 4).

**Table 3:** All-cause mortality according to smoking exposure at time of diagnosis among 5,892 women with invasive breast cancer (1987 to 2008)

**Smoking status**			**All-cause mortality**		
	**Number of**		**Crude HR (95% CI)**	**Age**^**a**^**-adjusted HR (95% CI)**	**Adjusted**^**b **^**HR (95% CI)**
	**Women**	**Deaths**			
Never^c^	3,510	859	1.00	1.00	1.00
Former	1,303	267	0.93 (0.81 to 1.06)	1.10 (0.95 to 1.26)	1.17 (1.01 to 1.34)
Current^d^	1,079	282	1.00 (0.87 to 1.14)	1.21 (1.05 to 1.39)	1.38 (1.20 to 1.60)
≤15 pack years	232	52	0.67 (0.51 to 0.89)	0.77 (0.58 to 1.02)	0.89 (0.67 to 1.19)
>15 to ≤30 pack years	278	88	1.13 (0.91 to 1.41)	1.37 (1.09 to 1.71)	1.62 (1.29 to 2.03)
>30 pack years	273	93	1.30 (1.05 to 1.61)	1.62 (1.30 to 2.01)	1.83 (1.47 to 2.29)
P-value, test for trend^e^			0.0003	< 0.0001	< 0.0001
≤10 cigarettes/day	287	64	0.89 (0.69 to 1.15)	1.01 (0.79 to 1.31)	1.23 (0.95 to 1.60)
>10 to ≤20 cigarettes/day	395	114	1.04 (0.86 to 1.27)	1.24 (1.02 to 1.51)	1.36 (1.11 to 1.67)
>20 cigarettes/day	337	97	1.08 (0.87 to 1.33)	1.41 (1.14 to 1.75)	1.56 (1.26 to 1.94)
P-value, test for trend^e^			0.30	0.001	0.0001
≤20 years	191	54	0.80 (0.61 to 1.06)	0.88 (0.66 to 1.17)	1.00 (0.75 to 1.34)
21 to 30 years	258	66	0.81 (0.63 to 1.04)	1.06 (0.82 to 1.38)	1.24 (0.95 to 1.61)
>30 years	340	115	1.45 (1.20 to 1.77)	1.65 (1.36 to 2.01)	1.95 (1.59 to 2.38)
P-value, test for trend^e^			0.002	< 0.0001	< 0.0001

^a^HRs adjusted for age at diagnosis (≤39, 40 to 49, 50 to 59, 60 to 69, ≥70 years). ^b^HRs adjusted for age at diagnosis (≤39, 40 to 49, 50 to 59, 60 to 69, ≥70 years), year of diagnosis (≤1994, 1995 to 1999, 2000 to 2004, ≥2005), age at menarche (≤11, 12, 13, ≥14 years), parity (0, 1, 2, ≥3), menopausal status (premenopausal, postmenopausal), current hormone replacement therapy use (yes, no), first degree family history of breast cancer (yes, no), estrogen and progesterone receptors positivity (positive, negative, not investigated), histological grade (well, moderately, poorly differentiated), size of the tumor (≤10, 11 to 20, 21 to 30, 31 to 40, >40 mm, no pathological investigation), regional or distant involvement (node-negative, 1 to 3 positive nodes, ≥4 positive nodes, unknown nodal involvement, distant metastases), locoregional treatment (mastectomy, breast-conserving surgery, no surgery), neoadjuvant therapy (yes, no), adjuvant endocrine therapy (yes, no), adjuvant chemotherapy (yes, no). ^c^Reference category. ^d^Among the 1,079 current smokers, number of pack years of smoking, number of cigarettes smoked and duration of smoking were unknown for 296, 60 and 290 women, respectively. ^e^*P* for trend in hazard ratios comparing current smokers further categorized based on number of pack years of smoking, number of cigarettes smoked daily or duration of smoking, to never smokers. HR, hazard ratio; CI, confidence interval.

### Systematic review and meta-analysis of published findings

**Figure 1:**
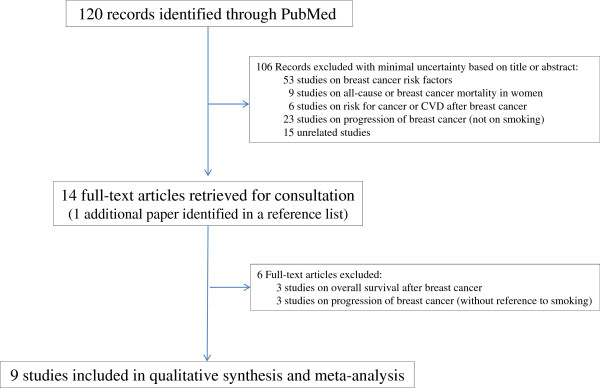
Search strategy and study selection process used in the meta-analysis of the association between smoking status and breast cancer-specific mortality.

A total of 120 records were identified through PubMed searching and no duplicate was found (Figure 1). Of the 120 records screened, the two reviewers independently identified the same nine published relevant studies on the relation of smoking to breast cancer-specific survival [4-6,8,9,11,12,15,16]; these studies are summarized in Table 4 (which includes the present study). Four [9,11,12,16] of these studies were reported in the review identified on smoking and breast cancer prognosis published in 2009 [3]; the study from Japan [15] was not included in the 2009 review, and four studies were published after the publication of the 2009 review [4-6,8]. A recent systematic review published in 2012 [5] included only five of the ten studies in Table 4. To our knowledge, no meta-analysis of smoking at diagnosis and post-diagnosis breast cancer specific mortality has been published yet.

**Table 4:** Studies included in the meta‒analysis of the association between smoking status and breast cancer‒specific mortality

	**Country**	**Years of follow-up**	**Stage**	**No. participants**		**Breast cancer deaths**	
**Study**				**Current**	**Never**	**Current**	**Never**
				**smokers**	**smokers**	**smokers**	**smokers**
Tominaga *et al*. [15]	Japan	1986 to 1995	I to IV	68	325	-	-
Manjer *et al*. [12]	Sweden	1977 to 1996	0 to IV	216	491	48	81
Fentiman *et al*. [11]	UK	1984 to 2004	I to II	40	67	-	-
Sagiv *et al*. [16]	USA	1996 to 2002	Invasive	252	568	28	52
Holmes *et al*. [9]	USA	1978 to 2002	I to III	1,018	2,112	216	357
Dal Maso *et al*. [8]	Italy	1991 to 2006	I to IV	290	934	80	248
Hellmann *et al*. [6]	Denmark	1976 to 2007	Loc/reg/met	212	120	96	48
Braithwaite *et al*. [5]	USA	1997 to 2012	I to III	173	1194	25	111
Warren *et al*. [4]	USA	1982 to 2010	Loc/reg/met	143	480	-	-
Bérubé *et al*.	Canada	1987 to 2008	Loc/reg/met	1,079	3,510	185	583

Loc/reg/met, Local, regional and metastatic disease.

Overall, cases of breast cancer were diagnosed between 1976 and 2012. All except two studies [15,16] had at least 15 years of potential follow-up. The present study is the largest with 5,892 cases; the number of breast cancer cases ranged from 150 to 5,056 in the other studies. When reported, mean or median age of participants at diagnosis ranged between 54 and 67 years [5,6,8,9,11,12]. Most studies (including the present study) included invasive breast cancer cases of any stage [4,6,8,12,15,16] and three studies excluded cases of advanced disease at diagnosis [5,9,11]; one study also included cases of *in situ* breast cancer (representing 12% of all cases) [12].

The assessment of smoking was based on information reported by patients at the time of or shortly after diagnosis of breast cancer (as in the present study) [4,11,12,15,16], in the year following diagnosis [8], in the two years preceding the diagnosis (Nurses’ Health Study, NHS) [9], several years before diagnosis (mean 6.7 years before diagnosis) [6] or in the three years following diagnosis [5]. The proportion of current smokers ranged between 8% and 27% ([4,5,8,9,11,12,15,16] and the present study) but reached 50% in one study [6]. All studies measured smoking at one time only. No study assessed the effect of smoking cessation or variation in smoking habit over time after breast cancer diagnosis.

Deaths were identified through linkage to national registries, and cause of death from death certificates (as in the present study) [4,5,12,16] supplemented as needed by reviewing medical records [8], or from medical records only [11,15]. In the NHS (2007) [9], deaths were usually reported by families or postal authorities and supplemented with a search in the National Death Index, and cause of death was based on death certificates. The percentage of deaths attributable to breast cancer was 42% [12], 50% [5], 59% [16], 65% [9], 68% (the present study), 79% [8] and 100% [15].

All HRs are minimally adjusted for age at diagnosis and stage (except in [16]). The HRs in [6,9] and in the present study are also adjusted for body mass index (BMI). The HRs in [8,9] and in the present study are also adjusted for year of diagnosis; women were recruited in only one calendar year in [16]. All HRs except one [12] are additionally adjusted for potential confounders other than age, stage, BMI or year of diagnosis.

**Figure 2:**
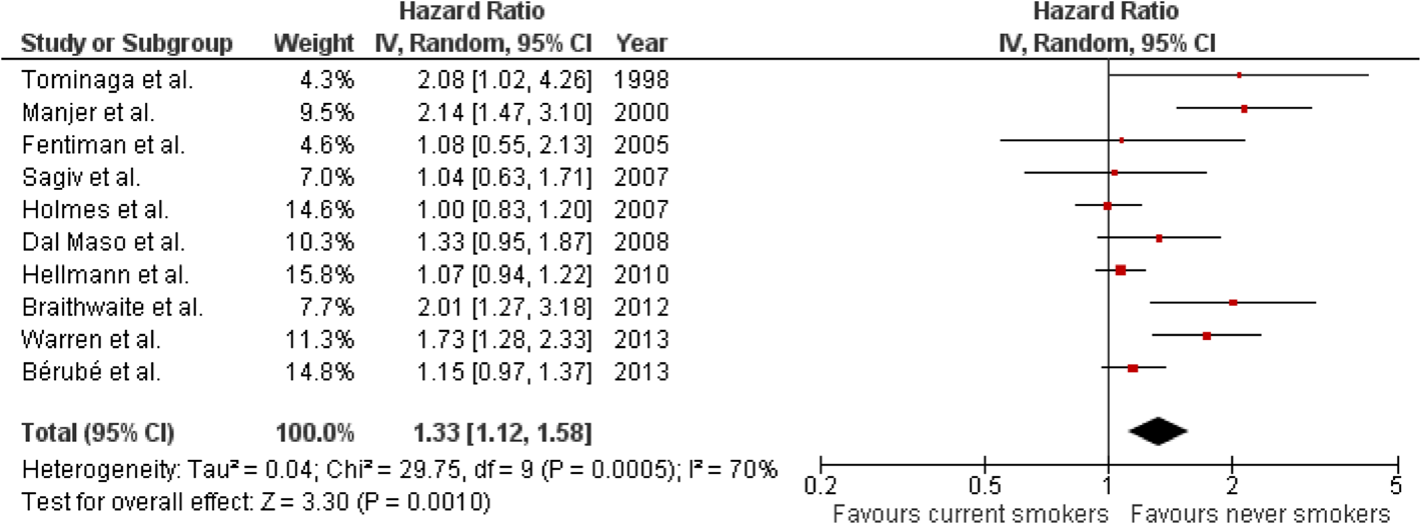
**Forest plot with study specific and random effects for association between smoking status (current versus never) and breast cancer specific-mortality.** In two studies, the authors present multivariate adjusted HRs for current smokers from two distinct models (HRs of 1.41 and 1.08 in Table 2 in Fentiman [11] and HRs of 2.14 and 1.95 in Table 1 in Manjer [12]); the present meta-analysis is based on the HR that was adjusted for the most complete set of prognostic factors including age. In the study by Dal Maso *et al*. [8] results were published separately for cases currently smoking <15 cigarettes/day (HR: 1.39) and those smoking ≥15 cigarettes/day (HR: 1.23), as compared with never smokers; the corresponding HRs among current smokers were weighted in proportion to the numbers in each subgroup (182 and 108, respectively) and then combined. The summary HR and 95% CI are from random-effects models. CI, confidence interval; HR, hazard ratio.

**Figure 3:**
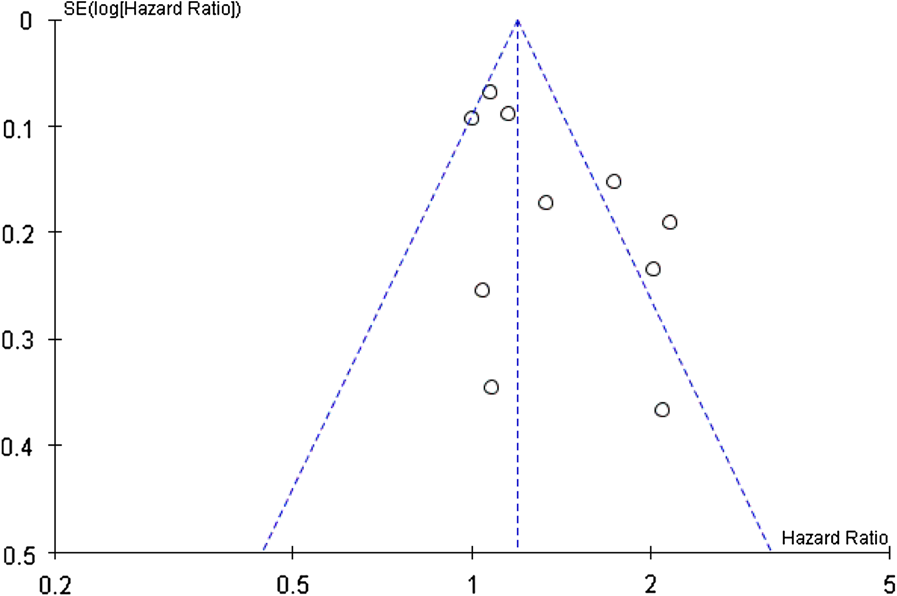
Funnel plot with a triangular 95% confidence region.

Results from available studies on smoking at diagnosis and breast cancer-specific mortality after diagnosis [4-6,8,9,11,12,15,16] are presented in Table 4 and Figure 2. The summary breast cancer-specific HR for current smokers as compared with never smokers is 1.33 (95% CI: 1.12 to 1.58); test for overall effect, random-effects model; *P* = 0.001). Based on the Q statistic (*P* = 0.0005) and the I^2^ index (70%), there was rather high [35] heterogeneity between studies (Figure 2 and Figure 3). After the exclusion of the three studies most likely responsible for heterogeneity [4,5,12], there was no more heterogeneity between studies (Q statistic: *P* = 0.45; I^2^ index = 0%), and the summary HR for current smokers at diagnosis as compared to never smokers is 1.10 (95% CI: 1.01 to 1.20). The test for overall effect was identical whether based on fixed-effects or random-effects models (*P* = 0.03). These three papers [4,5,12] are in our view of good quality.

## Discussion

In this large cohort of breast cancer patients, breast cancer-specific mortality increased with total, intensity or duration of smoking at diagnosis. Moreover, the meta-analysis of all studies identified in this systematic review shows a statistically significant increased breast cancer-specific mortality among current smokers compared to non-smokers at diagnosis. Thus, current evidence indicates that smoking at diagnosis is associated with a reduced survival from breast cancer itself. Moreover, among available studies on smoking at diagnosis and overall survival [4-14,16,36-38], most [4-14,16] - including our own - show that current smokers have lower overall survival compared to never smokers.

Smoking status at time of diagnosis is a strong indicator of continued smoking after diagnosis since, among smoking women with breast cancer, only 4% are reported to have stopped smoking post-diagnosis [39]. To our knowledge, no study has compared breast cancer specific survival of smokers at diagnosis who continue smoking to that of smokers at diagnosis who stop smoking. Moreover, if smoking cessation has an effect on post-diagnosis breast cancer specific mortality, the time required for this effect of smoking cessation to be seen needs to be examined.

Smoking is suspected to play a role in the initiation and growth of many cancers, including breast cancer. Experimental evidence is accumulating on the contribution of nicotine to tumor growth and metastasis [40]. The effects of nicotine might be mediated through the nicotinic acetylcholine receptors, which are expressed in human breast cancer cells and regulate diverse signaling pathways involved in cell proliferation, angiogenesis, apoptosis and also in metastatic dissemination of the primary tumor. Such findings suggest that smoking can affect the incidence as well as the course of cancer. Epidemiological findings suggest that smoking is associated with increased breast cancer incidence [41].

Many women diagnosed with breast cancer are smokers – 18% of women in this Canadian population and 15% to 20% of US cancer survivors [1,42]. Among such a large population of smokers, actions aimed at smoking cessation can be justified by the strong and widely accepted association of smoking with overall survival [43]. The present study supports the idea that smoking could also be detrimental in terms of survival from the disease itself – information which could represent an important additional incentive for smoking cessation among smoking women with breast cancer. We previously reported that some women with early breast cancer undertake healthful behavior changes on their own and that these changes are generally coherent with current guidelines for things such as diet and physical activity [44]. However, as mentioned above, only 4% of smoking women with breast cancer are reported to have stopped smoking post-diagnosis [39].

This study has strengths and weaknesses. First, in this, as in most previous studies on the subject, smoking exposure was self-reported at time of breast cancer diagnosis. Moreover, we have no information on passive smoking. Second, cause of death was based on death certificates. As a result, the accuracy of causes of death may be imperfect [45], but this is unlikely related to smoking status. Third, multivariate adjusted models took into account a large set of potential confounders, including the main breast cancer prognostic factors. Nevertheless, residual confounding is possible and may be due, for instance, to unmeasured factors, such as mode of detection [46], delay in treatment [47], other lifestyle factors at the time of diagnosis (for example, physical activity [2,23]) or after diagnosis (for example, physical activity or weight control [2,3,23]). Finally, the present study is based on the experience of only one breast center. However, during the years 2002 to 2006, patients treated in this center represented 78% of all newly diagnosed invasive breast cancer cases among women living in this area. The study population is similar to Canadian women both with regard to smoking behavior at the time of diagnosis and to breast cancer survival. For instance, between 23% (1999) and 16% (2008) of Canadian women [48] and 18% (1987 to 2008) of the study population were current smokers. The five-year (87%) and ten-year (79%) breast cancer-specific survival estimates in the present study were similar to five-year (86% to 88% [49-51]) and ten-year (80% to 82% [49,50]) relative survival rate estimates available from Canadian national statistics.

Between-study heterogeneity that was apparent in the meta-analysis needs to be clarified. In our view, this heterogeneity is not explained by the quality of studies; almost all available studies were of good quality. However, the list of confounders taken into account in the analysis varies substantially from one study to the other. We cannot exclude the possibility of a publication bias.

## Conclusions

In summary, our new findings combined with those of previous studies indicate that smoking at diagnosis is associated with a reduction of breast cancer-specific survival as well as overall survival. The possibility that smoking cessation could improve survival from breast cancer itself needs to be assessed. If smoking after diagnosis was shown to be detrimental in terms of survival from the disease itself, this would represent an important additional incentive for smoking cessation efforts among smoking women with breast cancer and among physicians who care for them.

## Abbreviations

CI: confidence interval; CMSDF: Centre des maladies du sein Deschênes-Fabia; HR: hazard ratio; ISQ: Institut de la statistique du Québec; Loc/reg/met: local, regional and metastatic disease; NHS: Nurses’ Health Study.

## Competing interests

The authors declare that they have no competing interests.

## Authors’ contributions

SB participated in the design of the study, coordinated the data acquisition, planned statistical analysis with input from JB and LM, and wrote the first draft of the manuscript. JL participated in acquisition of data, assisted in interpretation of findings and was involved in critically revising the manuscript. LM assisted in development of the methodology and was involved in critically revising the manuscript. EM helped to draft the manuscript and assisted in the interpretation of findings. JB conceived of the study, participated in statistical analysis, in the interpretation of findings and in the drafting and revising of the manuscript. All authors read and approved the final manuscript.

## Supplementary Material

Additional file 1 **Complete case analysis of breast cancer-specific mortality according to smoking exposure at time of diagnosis among 4,334 women with invasive breast cancer (1987 to 2008).** Sensitivity analysis based on complete case analysis. Click here for file

Additional file 2 **Breast cancer-specific mortality according to smoking exposure at time of diagnosis among 5,892 women with invasive breast cancer (1987 to 2008) - Analysis with indicator variables for missing data on confounders.** Sensitivity analysis using missing indicator variables for confounders. Click here for file

Additional file 3 **Complete case analysis of all-cause mortality according to smoking exposure at time of diagnosis among 4,334 women with invasive breast cancer (1987 to 2008).** Sensitivity analysis based on complete case analysis. Click here for file

Additional file 4 **All-cause mortality according to smoking exposure at time of diagnosis among 5,892 women with invasive breast cancer (1987 to 2008) - Analysis with indicator variables for missing data on confounders.** Sensitivity analysis using missing indicator variables for confounders. Click here for file
