# Supplementary material for: Smoking at time of diagnosis and breast cancer-specific survival: new findings and systematic review with meta-analysis
Source: Breast Cancer Res. 2014 Apr 19;16(2):R42. doi: 10.1186/bcr3646 (PMC4053238; doi:10.1186/bcr3646)
Supplement: Additional file 1 — Complete case analysis of breast cancer-specific mortality according to smoking exposure at time of diagnosis among 4,334 women with invasive breast cancer (1987 to 2008). Sensitivity analysis based on complete case analysis. [file bcr3646-S1.pdf]

Additional file 1 Complete case analysis of breast cancer-specific mortality according to smoking exposure at time of diagnosis among 4,334 women with invasive breast cancer (1987-2008)

Smoking status	Number of Women	Number of Deaths	Breast cancer-specific mortality: Crude HR (95% CI)	Age[a]-adjusted HR (95% CI)	Adjusted[b] HR (95% CI)
Never[c]	2,517	401	1.00	1.00	1.00
Former	986	126	0.89 (0.73-1.09)	0.94 (0.77-1.15)	0.99 (0.81-1.22)
Current[d]	831	138	1.00 (0.82-1.21)	0.98 (0.81-1.20)	1.11 (0.91-1.36)
≤15 pack years	177	28	0.77 (0.53-1.14)	0.69 (0.47-1.02)	0.84 (0.57-1.25)
>15 to ≤30 pack years	218	43	1.11 (0.81-1.53)	1.07 (0.78-1.47)	1.18 (0.85-1.63)
>30 pack years	212	42	1.18 (0.86-1.62)	1.32 (0.96-1.82)	1.45 (1.05-2.02)
P-value, test for trend[e]			0.10	0.01	0.01
≤10 cigarettes/day	209	28	0.85 (0.58-1.25)	0.82 (0.56-1.21)	1.07 (0.72-1.57)
>10 to ≤20 cigarettes/day	313	54	0.98 (0.74-1.30)	0.98 (0.73-1.30)	1.07 (0.79-1.43)
>20 cigarettes/day	258	53	1.22 (0.91-1.62)	1.21 (0.90-1.61)	1.20 (0.89-1.62)
P-value, test for trend[e]			0.13	0.14	0.28
≤20 years	150	32	1.00 (0.70-1.44)	0.82 (0.56-1.18)	0.91 (0.62-1.33)
21 to 30 years	196	36	0.94 (0.67-1.32)	0.89 (0.63-1.26)	1.03 (0.72-1.46)
>30 years	265	46	1.13 (0.83-1.53)	1.33 (0.98-1.81)	1.54 (1.12-2.12)
P-value, test for trend[e]			0.57	0.08	0.009

Abbreviations: HR, hazard ratio; CI, confidence interval.

a HRs adjusted for age at diagnosis (≤39, 40-49, 50-59, 60-69, ≥70 years).

b HRs adjusted for age at diagnosis (≤39, 40-49, 50-59, 60-69, ≥70 years), year of diagnosis (≤1994, 1995-1999, 2000-2004, ≥2005), age at menarche (≤11, 12, 13, ≥14 years), parity (0, 1, 2, ≥3), menopausal status (premenopausal, postmenopausal), current hormone replacement therapy use (yes, no), first degree family history of breast cancer (yes, no), estrogen and progesterone receptors positivity (positive, negative, not investigated), histological grade (well, moderately, poorly differentiated), size of the tumor (≤10, 11-20, 21-30, 31-40, >40 mm, no pathological investigation), regional or distant involvement (node-negative, 1-3 positive nodes, ≥4 positive nodes, unknown nodal involvement, distant metastases), locoregional treatment (mastectomy, breast-conserving surgery, no surgery), neoadjuvant therapy (yes, no), adjuvant endocrine therapy (yes, no), adjuvant chemotherapy (yes, no).

c Reference category.

d Among the 1,079 current smokers, number of pack years of smoking, number of cigarettes smoked and duration of smoking were unknown for 296, 60 and 290 women, respectively.

e P for trend in hazard ratios comparing current smokers further categorized based on number of pack years of smoking, number of cigarettes smoked daily or duration of smoking, to never smokers.
