# Supplementary material for: Smoking at time of diagnosis and breast cancer-specific survival: new findings and systematic review with meta-analysis
Source: Breast Cancer Res. 2014 Apr 19;16(2):R42. doi: 10.1186/bcr3646 (PMC4053238; doi:10.1186/bcr3646)
Supplement: Additional file 2 — Breast cancer-specific mortality according to smoking exposure at time of diagnosis among 5,892 women with invasive breast cancer (1987 to 2008) - Analysis with indicator variables for missing data on confounders. Sensitivity analysis using missing indicator variables for confounders. [file bcr3646-S2.pdf]

Additional file 2 Breast cancer-specific mortality according to smoking exposure at time of diagnosis among 5,892 women with invasive breast cancer (1987-2008) - Analysis with indicator variables for missing data on confounders

Smoking status	Number of Women	Number of Deaths	Breast cancer-specific mortality: Crude HR (95% CI)	Age[a]-adjusted HR (95% CI)	Adjusted[b] HR (95% CI)
Never[c]	3,510	583	1.00	1.00	1.00
Former	1,303	185	0.94 (0.79-1.11)	0.99 (0.84-1.17)	1.06 (0.90-1.26)
Current[d]	1,079	185	0.99 (0.84-1.16)	0.99 (0.83-1.17)	1.15 (0.96-1.36)
≤15 pack years	232	40	0.82 (0.60-1.13)	0.73 (0.53-1.01)	0.94 (0.67-1.30)
>15 to ≤30 pack years	278	54	1.06 (0.80-1.40)	1.05 (0.79-1.39)	1.24 (0.93-1.65)
>30 pack years	273	58	1.20 (0.91-1.57)	1.35 (1.03-1.78)	1.52 (1.15-2.01)
P-value, test for trend[e]			0.08	0.004	0.002
≤10 cigarettes/day	287	36	0.75 (0.54-1.05)	0.73 (0.52-1.02)	0.98 (0.70-1.38)
>10 to ≤20 cigarettes/day	395	75	1.05 (0.83-1.34)	1.06 (0.83-1.35)	1.15 (0.89-1.47)
>20 cigarettes/day	337	70	1.17 (0.91-1.50)	1.19 (0.92-1.53)	1.28 (0.99-1.66)
P-value, test for trend[e]			0.06	0.04	0.05
≤20 years	191	44	1.06 (0.78-1.44)	0.87 (0.63-1.19)	1.04 (0.75-1.43)
21 to 30 years	258	49	0.93 (0.69-1.24)	0.92 (0.68-1.24)	1.10 (0.81-1.49)
>30 years	340	61	1.11 (0.85-1.45)	1.30 (1.00-1.70)	1.57 (1.19-2.06)
P-value, test for trend[e]			0.67	0.07	0.003

Abbreviations: HR, hazard ratio; CI, confidence interval.

a HRs adjusted for age at diagnosis (≤39, 40-49, 50-59, 60-69, ≥70 years).

b HRs adjusted for age at diagnosis (≤39, 40-49, 50-59, 60-69, ≥70 years), year of diagnosis (≤1994, 1995-1999, 2000-2004, ≥2005), ≥30.0), age at menarche (≤11, 12, 13, ≥14 years), parity (0, 1, 2, ≥3), menopausal status (premenopausal, postmenopausal), current hormone replacement therapy use (yes, no), first degree family history of breast cancer (yes, no), estrogen and progesterone receptors positivity (positive, negative, not investigated), histological grade (well, moderately, poorly differentiated), size of the tumor (≤10, 11-20, 21-30, 31-40, >40 mm, no pathological investigation), regional or distant involvement (node-negative, 1-3 positive nodes, ≥4 positive nodes, unknown nodal involvement, distant metastases), locoregional treatment (mastectomy, breast-conserving surgery, no surgery), neoadjuvant therapy (yes, no), adjuvant endocrine therapy (yes, no), adjuvant chemotherapy (yes, no).

c Reference category.

d Among the 1,079 current smokers, number of pack years of smoking, number of cigarettes smoked and duration of smoking were unknown for 296, 60 and 290 women, respectively.

e P for trend in hazard ratios comparing current smokers further categorized based on number of pack years of smoking, number of cigarettes smoked daily or duration of smoking, to never smokers.
