# Supplementary material for: Smoking at time of diagnosis and breast cancer-specific survival: new findings and systematic review with meta-analysis
Source: Breast Cancer Res. 2014 Apr 19;16(2):R42. doi: 10.1186/bcr3646 (PMC4053238; doi:10.1186/bcr3646)
Supplement: Additional file 3 — Complete case analysis of all-cause mortality according to smoking exposure at time of diagnosis among 4,334 women with invasive breast cancer (1987 to 2008). Sensitivity analysis based on complete case analysis. [file bcr3646-S3.pdf]

Additional file 3 Complete case analysis of all-cause mortality according to smoking exposure at time of diagnosis among 4,334 women with invasive breast cancer (1987-2008)

Smoking status	Number of Women	Number of Deaths	All-cause mortality Crude HR (95% CI)	Age[a]-adjusted HR (95% CI)	Adjusted[b] HR (95% CI)
Never[c]	2,517	575	1.00	1.00	1.00
Former	986	176	0.88 (0.74-1.04)	1.05 (0.88-1.24)	1.09 (0.91-1.29)
Current[d]	831	203	1.00 (0.85-1.17)	1.18 (1.00-1.39)	1.30 (1.10-1.54)
≤15 pack years	177	35	0.63 (0.45-0.88)	0.71 (0.50-1.00)	0.80 (0.56-1.14)
>15 to ≤30 pack years	218	69	1.20 (0.93-1.54)	1.39 (1.08-1.79)	1.55 (1.20-2.01)
>30 pack years	212	65	1.27 (0.98-1.64)	1.52 (1.18-1.97)	1.68 (1.29-2.18)
P-value, test for trend[e]			0.001	< 0.0001	< 0.0001
≤10 cigarettes/day	209	46	0.97 (0.72-1.30)	1.07 (0.79-1.45)	1.26 (0.93-1.71)
>10 to ≤20 cigarettes/day	313	82	0.99 (0.79-1.25)	1.15 (0.91-1.45)	1.26 (0.99-1.59)
>20 cigarettes/day	258	70	1.10 (0.86-1.41)	1.39(1.08-1.80)	1.40 (1.08-1.82)
P-value, test for trend[e]			0.45	0.01	0.02
≤20 years	150	39	0.77 (0.56-1.07)	0.85 (0.61-1.19)	0.91 (0.65-1.28)
21 to 30 years	196	48	0.83 (0.62-1.12)	1.02 (0.75-1.38)	1.17 (0.86-1.59)
>30 years	265	83	1.44 (1.15-1.82)	1.62 (1.29-2.05)	1.84 (1.45-2.34)
P-value, test for trend[e]			0.002	< 0.0001	< 0.0001

Abbreviations: HR, hazard ratio; CI, confidence interval.

a HRs adjusted for age at diagnosis (≤39, 40-49, 50-59, 60-69, ≥70 years).

b HRs adjusted for age at diagnosis (≤39, 40-49, 50-59, 60-69, ≥70 years), year of diagnosis (≤1994, 1995-1999, 2000-2004, ≥2005), age at menarche (≤11, 12, 13, ≥14 years), parity (0, 1, 2, ≥3), menopausal status (premenopausal, postmenopausal), current hormone replacement therapy use (yes, no), first degree family history of breast cancer (yes, no), estrogen and progesterone receptors positivity (positive, negative, not investigated), histological grade (well, moderately, poorly differentiated), size of the tumor (≤10, 11-20, 21-30, 31-40, >40 mm, no pathological investigation), regional or distant involvement (node-negative, 1-3 positive nodes, ≥4 positive nodes, unknown nodal involvement, distant metastases), locoregional treatment (mastectomy, breast-conserving surgery, no surgery), neoadjuvant therapy (yes, no), adjuvant endocrine therapy (yes, no), adjuvant chemotherapy (yes, no).

c Reference category.

d Among the 1,079 current smokers, number of pack years of smoking, number of cigarettes smoked and duration of smoking were unknown for 296, 60 and 290 women, respectively.

e P for trend in hazard ratios comparing current smokers further categorized based on number of pack years of smoking, number of cigarettes smoked daily or duration of smoking, to never smokers.
